# Brain mGluR5 in Shank3B^−/−^ Mice Studied With *in vivo* [^18^F]FPEB PET Imaging and *ex vivo* Immunoblotting

**DOI:** 10.3389/fpsyt.2019.00038

**Published:** 2019-02-12

**Authors:** Guohong Cai, Mengmeng Wang, Shuailiang Wang, Yi Liu, Yan Zhao, Yuanyuan Zhu, Suo Zhao, Ming Zhang, Baolin Guo, Han Yao, Wenting Wang, Jing Wang, Shengxi Wu

**Affiliations:** ^1^Department of Neurobiology, School of Basic Medicine, Fourth Military Medical University, Xi'an, China; ^2^Department of Nuclear Medicine, Xijing Hospital, Fourth Military Medical University, Xi'an, China; ^3^Department of Gastroenterology, First Affiliated Hospital of Xi'an Jiaotong University, Xi'an, China

**Keywords:** autism spectrum disorders, Shank3, PET, mGluR5, immunoblotting

## Abstract

Although several studies have found that metabotropic glutamate 5 receptor (mGluR5) may play an important role in autism spectrum disorders (ASD), the mechanisms remain unclear. Here, we used a Shank3 gene complete knockout mouse model (Shank3B^−/−^) to explore the change in mGluR5 in the brain. To assess whether deletion of Shank3 in mice results in ASD-like behavior, we conducted a battery of behavioral experiments to characterize Shank3B^−/−^ mice, including repetitive grooming behavior tests, three-chamber tests and resident-intruder tests. Wild-type C57/BL6 and Shank3B^−/−^ mice underwent PET scans with [^18^F]FPEB, which was highly specific to mGluR5. Mouse brains were extracted post-scan, and mGluR5 protein levels were assessed by immunoblotting. The binding potential (BPnd) of mGluR5 was rich in the hippocampus, thalamus, striatum, and amygdala. More importantly, Shank3B^−/−^ mice showed significantly increased BPnd compared to the control mice in these brain regions. Immunoblotting revealed elevated mGluR5 levels in the hippocampus, thalamus, and amygdala but not in the striatum compared with control mice. These findings indicated that [^18^F]FPEB could visualize mGluR5 in the mouse brain. The deficiency of Shank3 can impair mGluR5 expression in multiple brain regions. Future work is also needed to understand the reasons for different results between *in vivo* PET and *ex vivo* immunoblotting.

## Introduction

Previous studies have found that metabotropic glutamate 5 receptor (mGluR5) may play an important role in autism spectrum disorders (ASDs). However, the mechanisms remain poorly understood. Genetic defects of SHANK3 (PROSAP2) are one of the most replicated findings in autism genetics (1, 2). Because mouse models can provide unique insights into the mechanisms underlying ASD, numerous lines of Shank3 isoform-specific mutant mice with deletions of different exons or point mutations have been reported (3–9). These studies have consistently demonstrated that the deletion of Shank3 in mice resulted in abnormal behaviors relevant to ASD. Among them, Shank3B^−/−^ mice showed obvious repetitive behaviors and social interaction deficits (4). Therefore, Shank3B^−/−^ mice were used as ASD animal models in the present study.

Shank proteins, which are composed of five protein-protein interaction domains, interact with more than 30 synaptic proteins, including cell adhesion proteins, cytoskeletal proteins, and ionotropic and metabotropic glutamate receptors (mGluRs) (10, 11). It is worth noting that the alteration of mGluR5 gene expression and function has been identified as a risk factor for ASD (12, 13). It has been reported that *in vitro* mGluR5 expression and function would be strongly affected when the expression level of Shank3 was downregulated (14). In addition, *in vivo* Shank3 deletion can impair mGluR5 functions (9, 10). To study the role of this protein further, we conducted *in vivo* positron emission tomography (PET) studies of mGluR5 binding using 3-18F-fluoro-5-(2-pyridinylethynyl)benzonitrile) ([^18^F]FPEB) in Shank3 knockout (KO) and control mice. [^18^F]FPEB is safe, well tolerated, and suitable for quantifying mGluR5 in humans (15–17). Since the results of PET might be inconsistent with the results of semiquantitative experiments *in vitro* (18, 19), we also performed *ex vivo* immunoblotting to further verify the characteristics of mGluR5 expression in Shank3 KO mice.

## Methods

### Animals

In the present study, we used Shank3B^−/−^ mice as ASD mouse models, which were obtained from Prof. Guoping Feng (4). Shank3B^−/−^ mice and their wild-type control littermates were obtained by breeding heterozygotes with a C57BL/6J background. The animals were kept in a temperature-controlled room (22–26°C) under a 12-h light/dark cycle with free access to food and water. To acquire accurate results, animals were only used once in each test. All tests were conducted from 4 to 10 p.m.

### Behavioral Tests

#### Repetitive Grooming Behavior

Habituated individual mice were introduced into a transparent box without a top (22 cm length × 22 cm width × 25 cm height), which was placed on a table with only the ceiling of the room visible to avoid the generation of fear. The testing room was lighted at ~40 lux. The front-mounted video camera was placed 1 m away from the box and recorded a 40-min session, which included the mouse being introduced into the box and the initial 10-min segment of habituation that was not scored. The components of a grooming event included forelimb movement, rubbing the face and then the flanks, and finally the tail and genitals. The cumulative time spent grooming and the total number of grooming events during the final 30-min test segment were calculated by an observer blinded to the genotype.

### The Three-Chamber Test

The test mouse was placed in the low-illuminated testing room for at least 1 h prior to the start of the experiment. A conspecific target mouse, matched for age and sex and unfamiliar to the test mouse, was habituated to being put inside a wire cage for 1 h each day for at least 5 days before the test. The social test apparatus was an opaque acrylic box with two pull-out doors and three chambers. Each chamber was identical in size (41 × 20 cm), with the dimensions of the entire box being 63 (length) × 43 (width) × 23 cm (height). There was a 10-cm gap between adjacent chambers that could be opened or closed with the removable doors. The transparent wire cage (12 cm in height and 9.5 cm wide) equipped with the novel, target mouse was placed 2 centimeters away from the edge of the testing chamber to allow an interaction between the mice.

The whole experiment was performed under low illumination and quiet conditions. The unfamiliar, target mouse was introduced into the wire cage in one side compartment, and an empty cage was placed in the opposite side compartment. The test mouse was introduced into the middle chamber and habituated for at least 5 min. The partitions were then removed, and the test mouse was permitted to explore all 3 compartments for 10 min. The entire process was recorded by a CCTV camera hanging 3 m above the apparatus. The relative positions of the empty cage and social cage were counterbalanced across test animals. The time spent in each compartment was recorded using the automated software SMART.

### Resident-Intruder Test

The test mouse was placed individually in the test room to habituate for 1 h before the start of the experiment. A smaller, same-sex mouse selected as the target mouse was distinguished from the test mouse during the calculation of social behavior. The animals were fed in isolation for 3 days before the test day to motivate social behavior. The test was recorded by a CCTV camera for 10 min after the target mouse was introduced into the home cage of the test mouse. The specific episodes included sniffing (e.g., nose-to-nose, anogenital sniffing) and moving away from, following and pushing each other. The duration and frequency of these episodes initiated by the test animal toward the intruder animal were measured by a well-trained experimenter blinded to the genotype of the mouse.

### PET Ligand and Imaging

PET imaging studies were conducted in six Shank3B^−/−^ mice and six control mice. The animals were anesthetized using isoflurane (1.0–1.5% with oxygen flow of 1–1.5 L/min), and a tail vein was catheterized for radiotracer injection. Animal physiology was monitored using a system included with the imaging device (InterView™ FUSION, Mediso). The center resolution of the field of view of the PET scanner was 0.7 mm. Radioactive [^18^F]FPEB (150–200 Ci) was injected via the tail vein. After radiotracer injection, dynamic volumetric data were acquired for 10 min. Anatomical maps and data for attenuation correction were obtained during the PET studies (19). The mouse brain-atlas template from the Laboratory of Neuro Imaging (LONI) was applied for segmentation of regions of interest (ROIs). The ROIs selected for analysis were whole brain, olfactory bulb, cortex, striatum, hippocampus, thalamus, amygdala, hypothalamus, and cerebellum. The data were analyzed using PMOD3.2 (PMOD, Zurich, Switzerland) by MITRO Biotech Co., Ltd. Binding potential (BPnd) was determined for mGluR5 using muscular tissue data as an input function.

### Western Blot Analysis

Brains were removed from Shank3B^−/−^ mice and WT mice and sectioned into 1-mm-thick coronal sections at the end of the study. The tissues of each brain area were extracted from the sections according to the mouse atlas (*The Mouse Brain in Stereotaxic Coordinates*, second edition, by George Paxinos and Keith B.J. Franklin). The collected tissues were lysed in 100–300 μL of RIPA lysis buffer (10 mM Tris, 150 mM NaCl, 1% Triton X-100, 0.5% NP-40, and 1 mM EDTA at pH 7.4) containing a 1:100 (v/v) ratio of a protease inhibitor cocktail and a phosphatase inhibitor cocktail (Roche). We used the bicinchoninic acid protein assay (Pierce) to quantify total protein samples (20–40 μg). Then, the samples were resolved via sodium dodecyl sulfate–polyacrylamide gel electrophoresis (SDS-PAGE) and transferred to polyvinylidene fluoride membranes. The primary antibodies were as follows: anti-β-actin (1:1,000, Cell Signaling Technology); anti-Shank3 (1:1,000, Abcam); anti-mGluR5 (1:1,000, Abcam); anti-NR2b (1:1,000, Cell Signaling Technology); and anti-homer1 (1:1,000, Abcam). All western blots were visualized using the enhanced chemiluminescence detection method (Advansta). The scanned images were quantified using ImageJ software (version 1.47).

### Statistical Analyses

The data were analyzed with SPSS 21 (SPSS Inc., Chicago, IL, USA) or GraphPad Prism 7.0 and were expressed as the mean ± s.e.m. Comparisons between Shank3B^−/−^ mice and control mice without regard to sex were conducted with the independent *t*-test or two-way analysis of variance (ANOVA).

## Results

### Shank3B^−/−^ Mice Display Core Behavioral Features of ASDs

**Figure 1:**
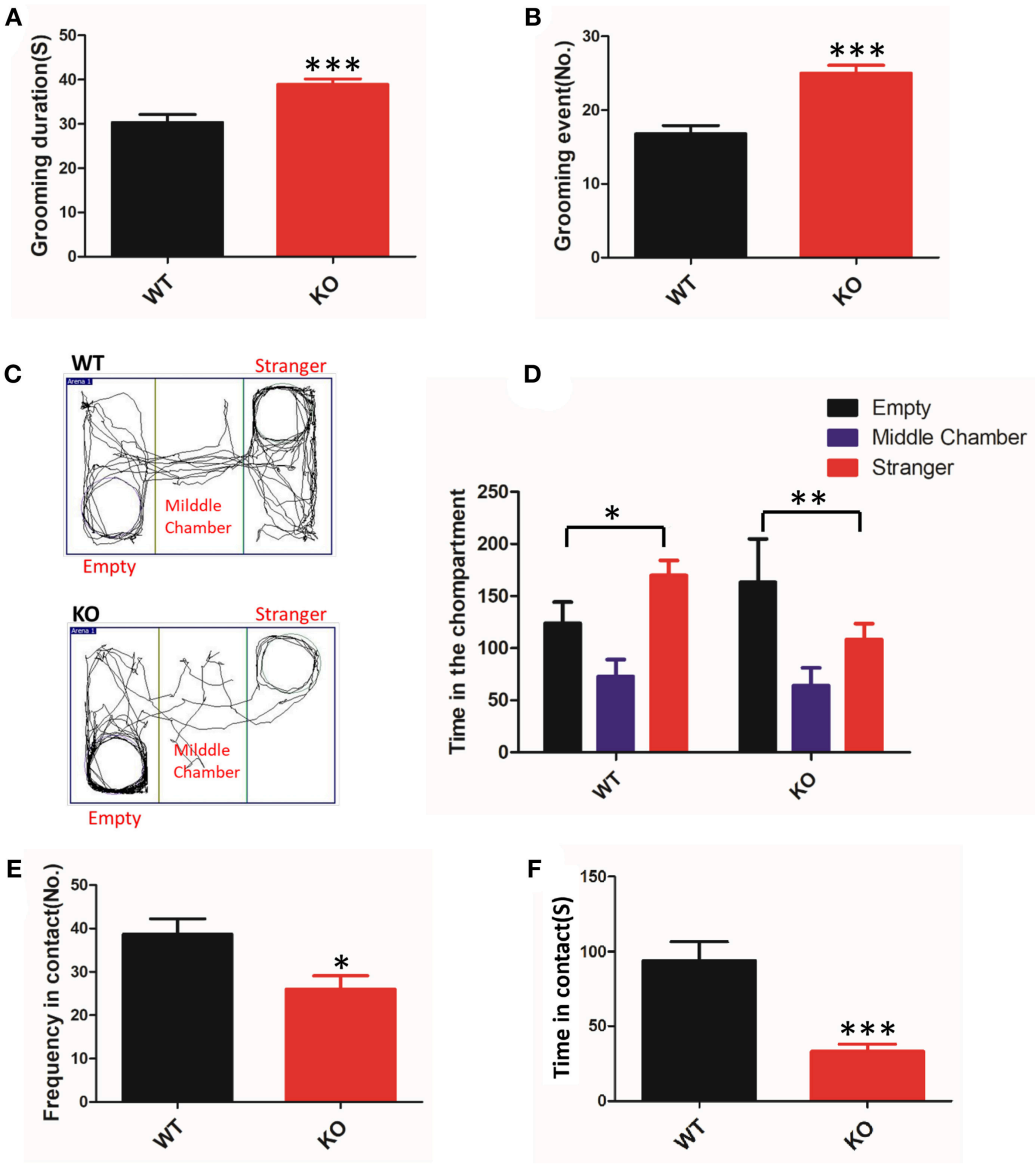
ASD-like behaviors in Shank3B^−/−^ mice. **(A,B)** Repetitive grooming was scored by the duration **(A)** and the total number of grooming events **(B)**. **(C)** Compared to the WT mice, the KO mice prefer to be in the chamber with the empty cage, as shown in the tracking map. **(D)** The KO mice spent more time in the chamber containing the empty cage and spent less time in the chamber associated with the unfamiliar mouse. **(E,F)** The resident-intruder interaction was evaluated by the frequency **(E)** and cumulative time of the social interactions **(F)**. The KO mice showed a clear reduction in social contact. ^*^p < 0.05, ^**^p < 0.01, ^***^p < 0.0001, compared to littermate WT mice. All data are displayed as the mean ± s.e.m. of 10–13 mice per group. Student's *t*-test was used for **(A,B,E,F)**; two-way ANOVA with Bonferroni's *post hoc t*-test for **(C)** and **(D)** were conducted for the statistical analysis.

To clarify whether deletion of Shank3 in mice results in ASD-like behavior, we conducted a battery of behavioral experiments to characterize Shank3B^−/−^ mice. The grooming behaviors of animals were measured for analysis of repetitive stereotyped behaviors, as one of the core symptoms of ASD. We found that KO mice displayed a clear increase in time spent grooming and in the total number of grooming events compared with the WT mice (Figures 1A,B). Thus, Shank3B^−/−^ mice showed self-injurious and excessive grooming behavior.

To assess defective social interactions, another core symptom of ASD, we measured the instinctual reaction of social interaction using the three-chamber test. The test mouse was free to explore the apparatus, and the preference to contact the target mouse placed inside the wire cage vs. the empty cage placed in the opposite chamber was assessed. In the test, KO mice favored contact with the empty cage, whereas the WT mice remained closer to the chamber containing the novel mice (Figures 1C,D). The observed abnormal initiation of social interaction in Shank3B^−/−^ mice was an indicator of impairment.

In a subsequent trial, we tested the mice on social motivation using the resident-intruder test. Compared to the WT littermates, the KO mice showed a reduction in the time and frequency of social contact (Figures 1E,F). These data suggested that Shank3 mutant mice were indifferent in situations involving social interaction.

### [^18^F]FPEB Synthesis

**Figure 2:**
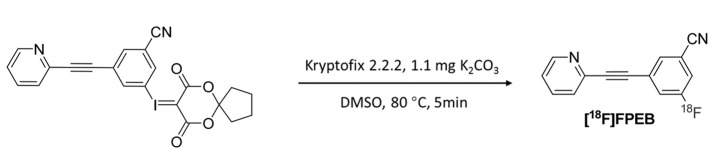
Synthesis of [^18^F]FPEB.

The radiotracer [^18^F]FPEB was prepared in an automated synthesis module, as described in a previous study (20) (Figure 2). The product was concentrated and rinsed with 10 mL of water. The final product was eluted with 3 mL of ethanol and collected into a product vial. The product solution was removed from the hot chamber and dried in a nitrogen blower. The solution was reconstituted with a small amount of ethanol, diluted with physiological saline, and sterilized by filtration through a 0.22-μm filter. The final product was in a sterile physiological saline solution with an ethanol concentration <7% (v/v).

### *In Vivo* mGluR5 Expression in Shank3B^−/−^ Mouse Brain

**Figure 3:**
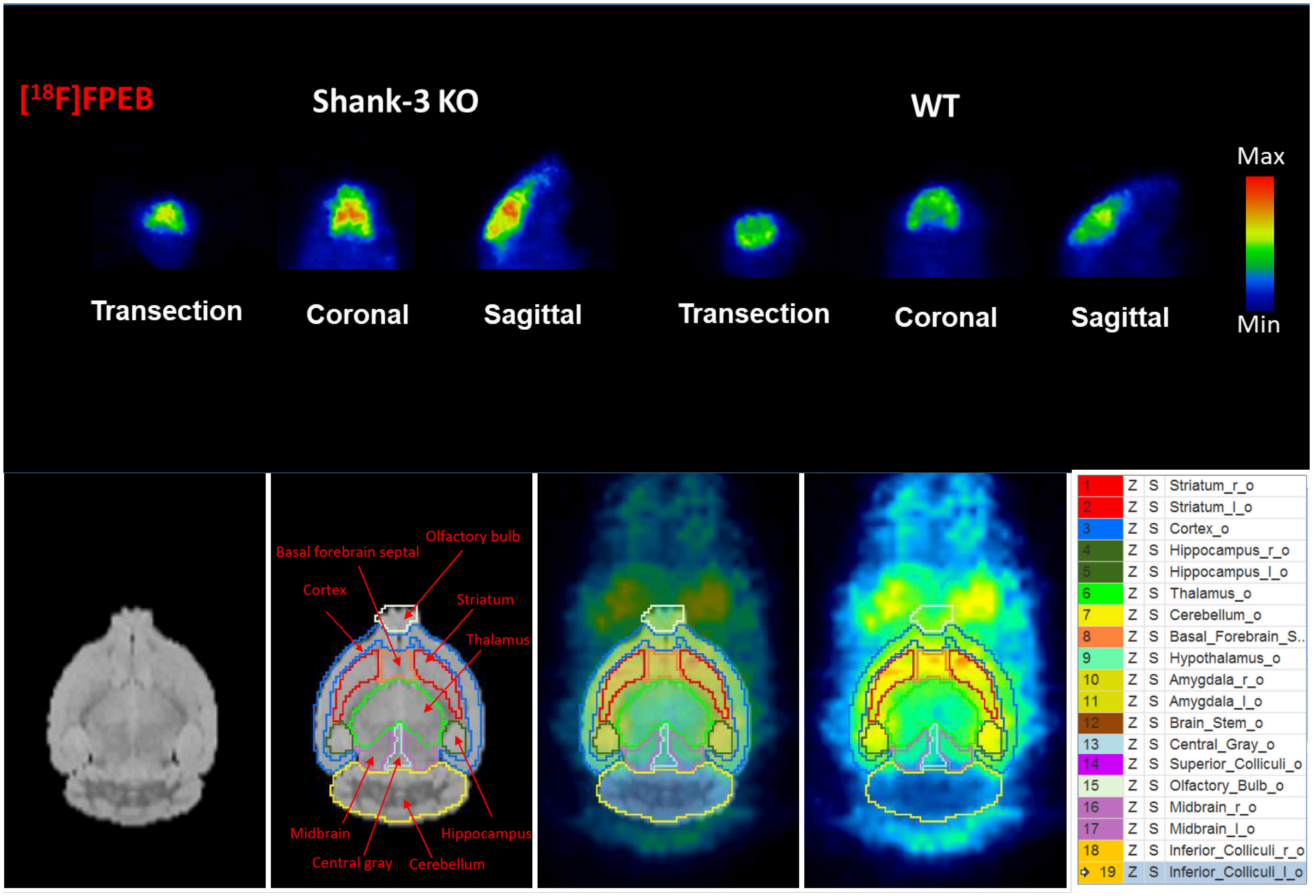
Binding of [^18^F]FPEB to Shank3B KO mice and their littermate control mice shows significant differences between the two groups.

**Figure 4:**
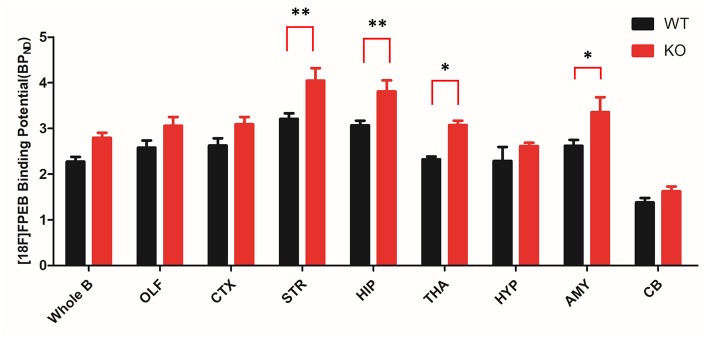
Bar graph of binding of [^18^F]FPEB to Shank3 KO (*n* = 6) and control mice (*n* = 6). Shank3 KO mice show significant increases in several brain regions compared to the control mice. STR and HIP: ^**^p < 0.01; THA and AMY: ^*^p < 0.05; whole brain, OLF, CTX, HYP, and CB showed no significant changes. OLF, olfactory bulb; CTX, cortex; STR, striatum; HIP, hippocampus; THA, thalamus; HYP, hypothalamus; AMY, amygdala; CB, cerebellum. ^*^p < 0.05, ^**^p < 0.01.

To investigate mGluR5 distribution in the brain, Shank3B^−/−^ mice and control mice were administered [^18^F]FPEB and PET-scanned for 10 min. PET data were quantified as binding potential (BPnd) in several brain regions by using the simplified tissue reference model with the muscular tissue as the reference region (Figure 3). The regions of interest included the olfactory bulb, cortex, striatum, hippocampus, thalamus, amygdala, hypothalamus, and cerebellum. The BPnd of mGluR5 was rich in the hippocampus, thalamus, striatum and amygdala (Figure 4). More importantly, Shank3B^−/−^ mice showed significantly increased BPnd compared to the control mice in the hippocampus (*P* < 0.01), striatum (*P* < 0.01), thalamus (*P* < 0.05), and amygdala (*P* < 0.05) (Figure 4).

### Deletion of Shank3 Reduces mGluR5 Expression in the Striatum

**Figure 5:**
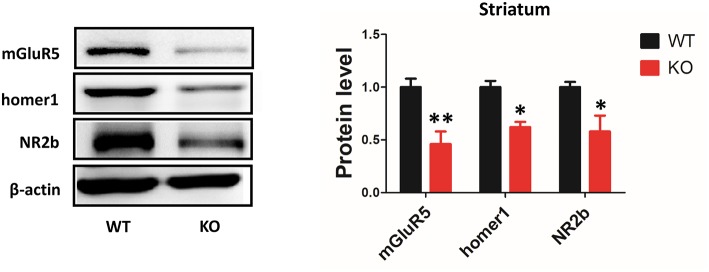
Deletion of Shank3 reduces mGluR5 expression in the striatum. mGluR5, homer1 and NR2b were reduced in the striatum from Shank3B^−/−^ mice. Each lane was loaded with 3 μg of protein, with β-actin as a loading control and normalized to wild-type levels. ^*^p < 0.05, ^**^p < 0.01, two-tailed *t*-test; all data are presented as the means ± s.e.m.; *n* = 3 samples per group.

It has been proposed that Shank3 plays an important role in forming excitatory synapses via its multiple protein-protein interactions (21). Shank proteins are indirectly connected to group I mGlu receptors by Homer proteins. A previous study has shown that the protein levels of the scaffolding proteins (SAPAP3, homer, and PSD93) and glutamate receptor subunits (GluR2, NR2A, and NR2B) were reduced in striatal PSD fractions from Shank3B^−/−^mice (4). However, the expression levels of mGluR5 in the striatum of Shank3B^−/−^ mutants remained unknown. Our data showed that mGluR5 protein level was reduced in the striatum from Shank3B^−/−^ mice. In addition, consistent with previous results, homer1 and NR2b were reduced in the striatum of Shank3B^−/−^ mice (Figure 5).

### mGluR5 Level Was Increased in Multiple Brain Regions of Shank3B^−/−^ Mice

**Figure 6:**
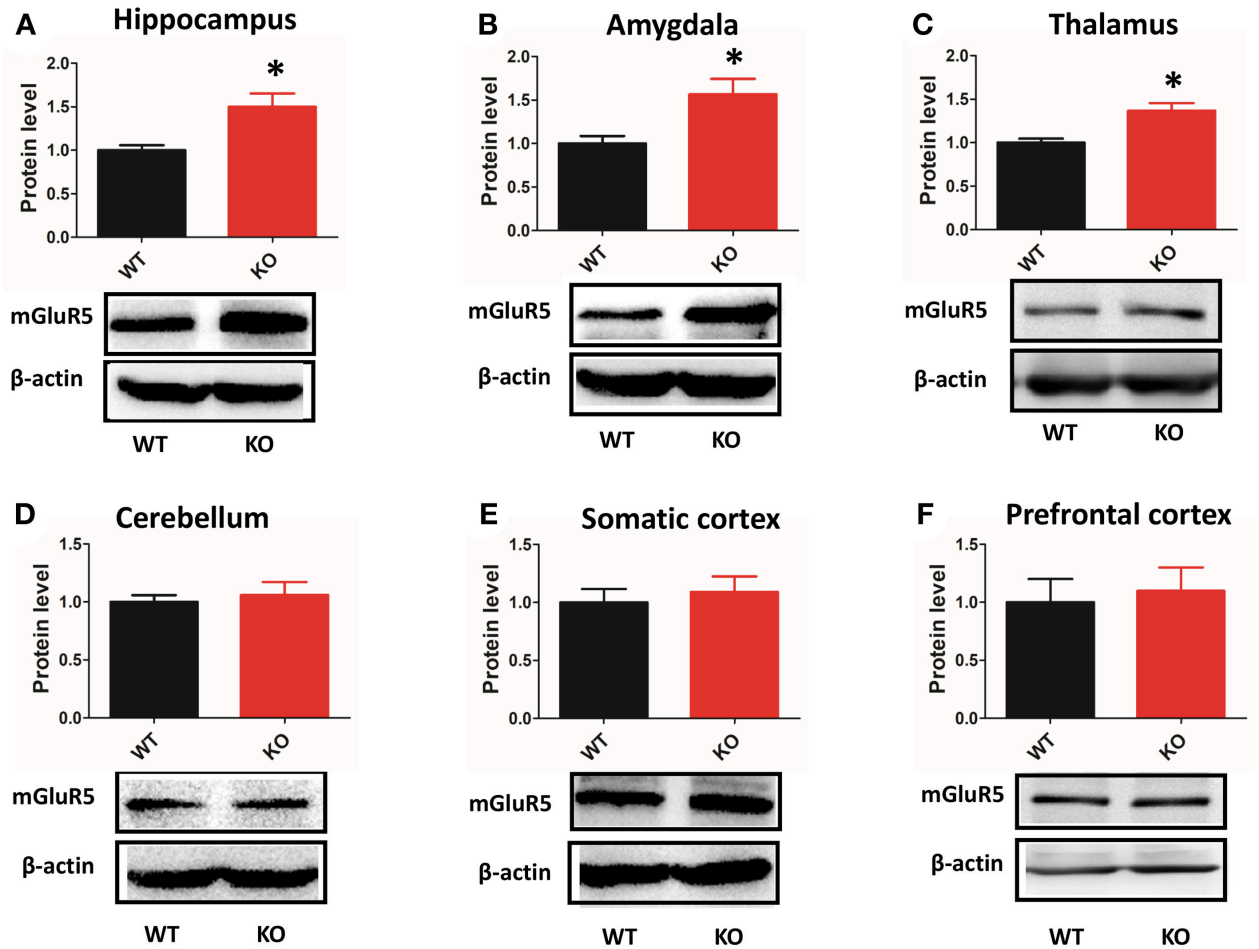
Protein levels of mGluR5 are increased in multiple brain regions of Shank3B^−/−^ mice. **(A–C)** mGluR5 is increased in the hippocampus, amygdala and thalamus of Shank3B^−/−^ mice. **(D–F)** mGluR5 level did not change in the cerebellum, somatic cortex or prefrontal cortex of Shank3B^−/−^ mice. Each lane was loaded with 3 μg of protein, with β-actin as a loading control and normalized to wild-type levels. ^*^p < 0.05, two-tailed *t*-test; all data are presented as the means ± s.e.m; *n* = 3 samples per group.

Depending on the brain region, Shank3 performed different functions at synapses (9, 10). Thus, we examined the expression of mGluR5 in multiple brain regions by western blot. mGluR5 was increased in the hippocampus, amygdala and thalamus of Shank3B^−/−^mice (Figures 6A–C). However, the level mGluR5 did not change in the cerebellum, somatic cortex or prefrontal cortex of Shank3B^−/−^ mice (Figures 6D–F).

## Discussion

The present study investigated the changes in mGluR5 expression in Shank3B^−/−^ mice and studied whether these changes could be imaged with the PET radioligand [^18^F]FPEB. Previous studies have pointed toward the involvement of mGluR5 in the pathological process resembling autism caused by the complete knockout of Shank3 (9, 10). Our study, to some extent, confirmed previous reports of differences in mGluR5 expression between Shank3 KO mice and wild-type mice.

To our knowledge, this was the first *in vivo* study of mGluR5 in the Shank3 KO mouse model. Previously, the interaction between mGluR5 and autism had been investigated in Shank3Δ11^−/−^ mice and Shank3Δ4-22^−/−^mice; however, these were all *ex vivo* experiments (9, 10). In an earlier study, Verpelli et al. used RNAi to knock down Shank3 expression in neuronal cultures that specifically reduced the synaptic expression of mGluR5 but did not affect the expression of other major synaptic proteins (14). In addition, the reduced mGluR5 activity in Shank3-knockdown neurons can be rescued by an allosteric agonist of mGluR5, such as CDPPB (14). However, subsequent study results from Shank3Δ11^−/−^ and Shank3Δ4-22^−/−^mice were wildly different (9, 10). Cinzia et al. found that the absence of Shank3 specifically reduced mGlu5/Homer interactions in the striatum and cortex, and the mGluR5 agonist CDPPB rescued ASD-like behavior in Shank3Δ11^−/−^ mice (10). In contrast, the study by Wang et al. showed a marked decrease in Homer1b/c and increased mGluR5 in the PSD fractions from the striatum of Shank3Δ4-22^−/−^mice, and some abnormal behaviors were normalized with the mGluR5 antagonist MPEP (9).

Our PET study showed high BPnd levels for [^18^F]FPEB in the hippocampus, thalamus and amygdala, which was consistent with the expression pattern of mGluR5 in the human brain (22). More importantly, increased mGluR5 expression has been observed in these brain regions of Shank3B^−/−^ mice compared to control mice. A recent pilot PET study, which also used [^18^F]FPEB as a tracer, showed increased binding potential in the postcentral gyrus and cerebellum of male individuals with autism (23).

Interestingly, the protein levels of mGluR5 assessed with immunoblotting could not be visualized with PET in any of the brain regions. For example, the protein level differences in mGluR5 in the striatum were not reflected in the changes in BPnd. This may be because of the limitation of performing PET on small animals. One such limitation is the spatial resolution of PET; the PET image resolution (1–3 mm) may not be sufficient for the small mouse brain (19).

In addition to the reduced protein level of mGluR5 in the striatum from Shank3B^−/−^ mice, our data showed that homer1 and NR2b were reduced in the striatum. These genes converge on the NMDA receptor complex (24, 25), which has itself been associated with ASD (26). More specifically, mGluR5 potentiates the NMDA receptor, while homer ensures the appropriate cell surface localization of the NMDAR/mGluR5 complex.

## Conclusion

[^18^F]FPEB appears to be a good tracer with high specificity for mGluR5 in the mouse brain. Our data acquired from postmortem tissue and PET indicated that the deficiency of Shank3 can impair the expression of mGluR5 to varying degrees in different brain regions. However, the result of PET was inconsistent with the result of western blot in the striatum. Future work is also needed in order to understand the reasons for the different results observed between *in vivo* PET and *ex vivo* immunoblotting.

## Ethics Statement

The experimental procedures were approved by the Animal Care and Use Committee of the FMMU and followed the National Institutes of Health Guide for the Care and Use of Laboratory Animals (NIH Publication No. 80-23, revised 1996).

## Author Contributions

SheW and JW conceived and designed the experiments. GC and ShuW performed most of the experiments and analyzed the data. GC and YL wrote and refined the article. MW, YuZ, BG, and HY participated in the animal modeling and behavioral experiments. YaZ, SZ, and MZ assisted in laboratory work and figure preparation. WW supervised the acquisition of results.

### Conflict of Interest Statement

The authors declare that the research was conducted in the absence of any commercial or financial relationships that could be construed as a potential conflict of interest.
